# Development and Validation of Vulnerable and Enabling Indices for HIV Viral Suppression among People with HIV Enrolled in the Ryan White Program

**DOI:** 10.3390/ijerph18137048

**Published:** 2021-07-01

**Authors:** Merhawi T. Gebrezgi, Mary Jo Trepka, Semiu O. Gbadamosi, Kristopher P. Fennie, Daisy Ramirez-Ortiz, Tan Li, Sofia B. Fernandez, Petra Brock, Robert A. Ladner, Diana M. Sheehan

**Affiliations:** 1 Department of Epidemiology, Robert Stempel College of Public Health and Social Work, Florida International University, 11200 SW 8th St., Miami, FL 33199, USA; mgebr003@fiu.edu (M.T.G.); trepkam@fiu.edu (M.J.T.); sgbadamo@fiu.edu (S.O.G.); daramire@fiu.edu (D.R.-O.); sofernan@fiu.edu (S.B.F.); 2 Research Centers in Minority Institutions (RCMI), Florida International University, 11200 SW 8th St., AHC 5, Room 479, Miami, FL 33199, USA; 3 Division of Natural Sciences, New College of Florida, 5800 Bayshore Rd, Sarasota, FL 34243, USA; kfennie@fiu.edu; 4 Department of Biostatistics, Robert Stempel College of Public Health and Social Work, Florida International University, 11200 SW 8th St., Miami, FL 33199, USA; tanli@fiu.edu; 5 Behavioral Science Research Corporation, 2121 Ponce de Leon Blvd, Coral Gables, FL 33134, USA; Petra_Brock@yahoo.com (P.B.); rladner@behavioralscience.com (R.A.L.); 6 Center for Substance Use and HIV/AIDS Research on Latinos in the United States (C-SALUD), Florida International University, 11200 SW 8th St., Miami, FL 33199, USA

**Keywords:** HIV, vulnerable factors, enabling factors, Behavioral Model for Vulnerable Populations, psychometric properties

## Abstract

Background: Numerous factors impact HIV care, often requiring consideration of indices to prevent collinearity when using statistical modeling. Using the Behavioral Model for Vulnerable Populations, we developed vulnerable and enabling indices for people living with HIV (PLWH). Methods: We used Ryan White Program (RWP) data and principal component analysis to develop general and gender- and racial/ethnic-specific indices. We assessed internal reliability (Cronbach’s alpha), convergent validity (correlation coefficient), and predictive utility (logistic regression) with non-viral suppression. Results: Three general factors accounting for 79.2% of indicators’ variability surfaced: mental health, drug use, and socioeconomic status (Cronbach’s alpha 0.68). Among the overall RWP population, indices showed convergent validity and predictive utility. Using gender- or racial/ethnic-specific indices did not improve psychometric performance. Discussion: General mental health, drug use, and socioeconomic indices using administrative data showed acceptable reliability, validity, and utility for non-viral suppression in an overall PLWH population and in gender- and racial/ethnic-stratified populations. These general indices may be used with similar validity and utility across gender and racial/ethnic diverse populations.

## 1. Introduction

The HIV care continuum, which includes diagnosis of HIV infection, linkage to and retention in HIV medical care, and maintenance of viral suppression, is a series of stages that people diagnosed with HIV go through to maintain their health and prevent HIV transmission [1,2]. In the United States, in 2016, among persons aged ≥ 13 living with HIV, 74.2% were engaged in care (defined as ≥1 CD4 or viral load test in 2016), 57.6% were retained in care (defined as having ≥2 tests ≥3 months apart in 2016), and 61.5% were virally suppressed (defined as viral load < 200 copies/mL on the last test in 2016) [3]. Identifying factors associated with the HIV care continuum outcomes is important to design intervention strategies and prevention policies.

In order to better understand HIV health services utilization and care outcomes among people living with HIV (PLWH), the Behavioral Model for Vulnerable Populations [4] was adapted to HIV care by Christopoulos et al. [5] and Ulett et al. [6]. In general, the model outlines individual and structural environment factors that determine the use of primary care resources by people with HIV. The individual factors include sociodemographic, vulnerable, enabling, and need factors that affect the continuum of HIV care [5,6]. Vulnerable factors include substance use and mental health factors that predispose the patient to using care, and enabling factors include economic and social factors that either enable or impede access to care.

Individual enabling and vulnerable factors have been found to predict HIV care continuum outcomes. These factors include drug use, homelessness, insurance status, transportation, housing, social support, income, immigration status, country of birth, sexual orientation, history of incarceration, mental health, and substance use [5,6,7,8,9,10,11,12]. To our knowledge, previous research has not developed and assessed the psychometric properties of vulnerable and enabling indices among PLWH, particularly using routinely collected administrative program data. One previous study developed HIV vulnerability indices among homeless persons [13]; however, these were not for HIV care and treatment.

Given the multitude of factors that may impact HIV care, it is often necessary to consider using indices that incorporate various factors into one construct to decrease correlation between variables when modeling their impact on HIV outcomes. Therefore, using the Behavioral Model for Vulnerable Populations [4,5,6] as a framework, the primary objective of this study was to develop vulnerable and enabling indices tailored for PLWH, and to assess the internal consistency, convergent validity, and predictive utility of these indices on HIV viral suppression. The secondary objective was to assess if gender- (men/women) and/or race/ethnicity-specific (Non-Hispanic Black, Hispanic, Haitian, Non-Hispanic White) vulnerable/enabling indices are needed.

## 2. Materials and Methods

### 2.1. Population

We used data from PLWH enrolled in the Miami-Dade County Ryan White Program (RWP) Part A/Minority AIDS Initiative (MAI) during the 2017 calendar year. The RWP provides medical care, medical case management and pharmaceutical, and other related support services to low income PLWH [14]. RWP Part A provides medical and support services to Eligible Metropolitan Areas (EMAs) and Transitional Grant Areas (TGAs) (counties/cities that are the most severely affected by the HIV/AIDS epidemic) [14]. The Minority AIDS Initiative supports clinician training, technical assistance, and the development of innovative strategies to improve access to HIV care and health outcomes for disproportionately affected minority populations, including black/African American populations [14].

Our study population included PLWH ≥ 18 years old enrolled in the RWP prior to January 2017, and who had received medical case management or peer education support network services during 2017. Clients who died in 2017, moved out of the county or state, became financially ineligible for the RWP, were incarcerated, or were dropped from the RWP due to no contact for at least 240 days, were excluded.

### 2.2. Vulnerable and Enabling Variables and Definition of Viral Suppression

Data were extracted from the 2016 and 2017 RWP comprehensive health assessment (needs assessment of RWP patients conducted twice a year), patient intake assessment (data collected at the time of entry to the RWP), and laboratory results reported to the RWP. We selected a total of 16 vulnerable- and enabling-related variables based on the Behavioral Model for Vulnerable Populations [4,5,6] and on their availability in the dataset. The variables considered for inclusion in the indices were mental health symptoms (client reporting feeling depressed or anxious); mental health services need (client received or needs mental health services); domestic violence (client ever experienced domestic violence); drug use (client reporting current intravenous or other illicit drug use); problematic drug use (assessed by three questions: (a) has drug use resulted in a hazardous situation, (b) has drug use resulted in legal problems, and (c) has drug use resulted in preventing client from carrying out daily activities), drug use affected adherence to antiretroviral therapy (ART)); substance use treatment need (client would like substance use treatment); lack of social support (client lacks social support system he/she can depend on); homelessness (client has non-permanent living arrangement including homeless, transient, or transitional housing); lack of transportation to appointments; food insecurity (client not getting the food he/she needs); perinatal HIV exposure (client reported HIV exposure due to perinatal transmission); injection drug use (IDU) exposure (client ever injected drugs or reported “IDU” or “IDU and male-to-male sexual intercourse” as mode of HIV transmission); number of minors in household (client reports one or more minors in household compared with zero minors); lack of HIV disclosure (client reports no disclosure of his/her HIV status to adult members of the family); and work status (client not currently working).

Non-viral suppression, the outcome used to validate the indices, was defined as having a viral load ≥ 200 copies/mL in the last viral load measurement in 2017.

### 2.3. Analysis

We developed the vulnerable/enabling indices by conducting reliability analysis and principal component analysis. First, we measured internal consistency using the Cronbach’s alpha. The impact of each variable on the overall scale was assessed by computing the Cronbach’s alpha when the respective variable was deleted. We deleted variables when the “Cronbach’s alpha if item deleted” improved (was higher).

Then, we conducted principal component analysis (PCA) with the remaining variables. In order to conduct PCA, we transformed the nominal variables by optimal scaling [15]. Then, we conducted nonlinear PCA to find the organizing principle of the variables. The number of factors to retain was decided based on an Eigenvalue greater than one [16]. The factors that led to a meaningful interpretation and made theoretical sense were ultimately selected. We deleted variables if their factor loading was <0.4 [17]. Finally, we computed scores for each person in our dataset, for each index, using the linear combination of standardized values of the variables in each factor. We used the same reliability and PCA methodology to create general indices (developed using data for the overall RWP client population) and gender- and racial/ethnic-specific indices (developed using stratified RWP client population by gender [men/women] and race/ethnicity [Non-Hispanic Black, Hispanic, Haitian, Non-Hispanic White/other]).

To evaluate convergent validity [18], we assessed the correlation between each index and non-viral suppression by calculating the point-biserial correlation coefficient (CC). Predictive validity (criterion-related validity) [19] was assessed by fitting crude logistic regression models, using non-viral suppression as the outcome. We fitted logistic regressions for the general indices on the outcome among the general population and for the general indices on the outcome among men, women, Non-Hispanic Black, Hispanic, Haitian, and Non-Hispanic White separately to assess if the general indices had similar predictive characteristics for each group. We also fitted logistic regression models for the group-specific indices to their respective group’s viral suppression status. Crude odds ratios and 95% confidence intervals were estimated. All analyses were conducted using SAS software V. 9.4 (SAS Institute, Cary, NC, USA).

This study was approved by the Florida International University Institutional Review Board.

## 3. Results

### 3.1. General Indices Developed Using the Overall RWP Client Population

Data from a total of 6939 RWP clients were used for developing and validating the indices. Seventy-six percent (5330) of clients were men. Approximately 57.5% (3989) were Hispanic, 24.9% (1731) Non-Hispanic Black, 10.8% (747) Haitian, and 6.8% (472) Non-Hispanic White. The proportion of all clients non-virally suppressed was 14.6% (1016). Non-viral suppression was higher for women than men (16.3% vs. 14.1%), and for Non-Hispanic Blacks (23.3%) than all other racial/ethnic groups (Hispanic: 10.6%; Haitian: 16.7%; Non-Hispanic White: 14.2%).

The final Cronbach’s Alpha of the general scale was 0.68 (Table 1). Six variables were excluded because removing them increased the Cronbach alpha by 0.11. The variables excluded were social support, transportation, perinatal HIV exposure, any IDU exposure, number of minors in the household, and HIV status disclosure. Two variables (food security and substance use treatment need) were excluded because their factor loading in the PCA step was <0.4. The first component included mental health symptoms, mental health services need and domestic violence and was labeled “mental health index”. The second component included drug use, problematic drug use, and adherence to ART when using drugs and was labeled “drug use index”. The third component included homelessness and current work status and was labeled “socioeconomic status index”. Higher scores corresponded to worsening mental health, drug use, and socioeconomic status. These three components accounted for 79.2% of the variability in the variables.

In the general indices, compared with the overall RWP population, men had lower (better) mean scores in the mental health (*p*-value = 0.0004) and socioeconomic (0.0035) indices, and women had higher (worse) scores in the mental health (<0.0001) and socioeconomic (<0.0001) indices. Men had lower (better) scores in the mental health (<0.0001) and socioeconomic (<0.0001) indices than women indicating men reported fewer mental health and socioeconomic challenges than women. Non-Hispanic Blacks had higher (worse) mean scores in the mental health (<0.0001), drug use (<0.0001), and socioeconomic (<0.0001) indices compared with the overall population. Hispanics had lower (better) mean scores in the mental health (<0.0001), drug use (<0.0001), and socioeconomic (<0.0001) indices compared with the overall population. Haitians had lower (better) mean scores in the mental health (<0.0001), and drug use (<0.0001) indices compared with the overall population. Non-Hispanic Whites had higher (worse) mean scores in the mental health (0.0007) and drug use (0.0189) indices compared with the overall population. Non-Hispanic Blacks had higher (worse) socioeconomic status scores than Non-Hispanic Whites (<0.0001). Hispanics and Haitians had lower (better) socioeconomic status (<0.0001), and drug use (<0.0001) scores compared with Non-Hispanic Whites. Hispanics additionally had lower (better) socioeconomic status scores (<0.0001) than Non-Hispanic Whites.

The point-biserial correlation coefficient between the general indices and non-viral suppression for the overall population were as follows: 0.11 (*p*-value < 0.0001) for the mental health index, 0.14 (*p*-value < 0.0001) for the drug use index, and 0.17 (*p*-value < 0.0001) for the socioeconomic status index (Table 2). All three general indices were significantly associated with non-viral suppression among the overall population; mental health (odds ratio (OR) 1.31, 95% confidence interval (CI): 1.24–1.39), drug use (OR 1.33, 95% CI: 1.26–1.40), and socioeconomic status (OR 1.50, 95% CI 1.42–1.59) (Table 2). The indices had similar predictive ability on the non-viral suppression status of males, females, Hispanics, Non-Hispanic Blacks, and Non-Hispanic Whites, except for the mental health and socioeconomic status indices for Haitians.

### 3.2. Indices by Gender and Race/Ethnicity

For the group-specific reliability analysis, in addition to the variables removed in the general analysis, domestic violence was removed for women and non-Hispanic Blacks (alpha increased from 0.5869 to 0.7071), and work status was removed for Haitians (alpha increased from 0.4745 to 0.5957) and Non-Hispanic Whites (alpha increased from 0.5881 to 0.7150) based on the Cronbach’s alpha if the item was deleted. However, substance use treatment need was included for Hispanics, Haitians, and Non-Hispanic Whites. After excluding these variables and those with factor loadings < 0.4, the Cronbach alpha in each group ranged from 0.66 to 0.72 (Table 1). PCA revealed few differences in factors developed for the overall population and the gender- and racial/ethnic-specific groups (Table 1). Similar to the general indices, the gender- and race/ethnicity-specific factors showed a mental health and a drug use construct. However, the factor surrounding socioeconomic status was unstable and included some drug use variables for women, Hispanics, Haitians, and Non-Hispanic Whites. The gender- and racial/ethnic-specific factors accounted for 59.1–85.3% of the variability in the variables.

The point-biserial correlation coefficient between the indices and non-viral suppression ranged from 0.13 to 0.19 for the men-specific indices, 0.08 to 0.16 for the women-specific indices, 0.10 to 0.14 for the Non-Hispanic Black-specific indices, 0.11 to 0.15 for the Hispanic-specific indices, 0.02 to 0.10 for the Haitian-specific indices, and 0.10 to 0.19 for the Non-Hispanic White-specific indices (Table 3). The group-specific indices significantly predicted non-viral suppression for the corresponding group, except for the mental health index for Haitians and the socioeconomic status index for Haitians and Non-Hispanic Whites, but the odds ratios were in the expected direction (Table 3).

## 4. Discussion

Our study has three main findings. First, our findings suggest that vulnerable and enabling factors for PLWH fall into three constructs: mental health, drug use, and socioeconomic status. Vulnerable/enabling variables had acceptable internal reliability, and factors correlated with and predicted non-viral suppression, although correlations and effect sizes were small. Second, we found that general indices developed with the overall population may be used for the overall population of PLWH in the RWP and for gender- and racial/ethnic-specific groups with similar validity and utility. Finally, the results suggest that while mental health and drug use constructs vary only slightly by gender and race/ethnicity, constructs related to socioeconomic status for these groups may differ significantly.

The three vulnerable/enabling constructs identified by our analysis, mental health, drug use, and socioeconomic status are consistent with constructs included in the Behavioral Model for Vulnerable Populations [4,5,6]. However, internal reliability of the vulnerable/enabling variables measured through the Cronbach’s alpha only reached 0.68, slightly lower than the typically preferred 0.70 cutoff [20], although others caution the use of cutoffs [21]. It is possible that some of the variables considered vulnerable and enabling indicators based on the Behavioral Model for Vulnerable Populations framework are actually part of a different overall construct among PLWH in the RWP, that the constructs are heterogenous, or that a larger number of variables were needed [22].

Despite only acceptable levels of internal reliability, the three indices developed were significantly correlated with and predicted non-viral suppression, suggesting some level of convergent validity and predictive utility. It is well known that mental health [23], substance use [24,25], and socioeconomic status [26] are associated with HIV care outcomes, including viral suppression among RWP clients [27,28].

Nevertheless, in our study, correlation coefficients were low [29] for all three indices and non-viral suppression, and the effect sizes in regression analyses were small [30]. It is possible that limitations regarding how the variables were measured affected convergent validity and predictive utility [31] as measurements were taken for service delivery purposes and validated instruments were not used.

The findings that vulnerable/enabling indices developed using the overall PLWH population had similar convergent validity and predictive utility in stratified analysis by gender and racial/ethnic group was encouraging. Together with the findings that gender- and racial/ethnic-specific indices do not have better reliability, convergent validity, or predictive utility, our study suggests that the vulnerable/enabling framework may function similarly across these populations, except for Haitian PLWH. Internal reliability of vulnerable/enabling variables by gender and racial/ethnic group was approximately 0.70 and similar to the reliability of the variables included in the analysis conducted with the overall population, except for Haitians where the reliability was <0.60. Mental health and substance use indices developed specifically for women/men and for each racial/ethnic group did not differ meaningfully from those developed for the overall population. Moreover, correlation coefficients and odds ratios for non-viral suppression remained small and did not strengthen when using gender- and racial/ethnic-specific factors compared with factors for the overall population. Interestingly, the mental health and substance use indices developed using the Haitian stratified population were not significantly associated with non-viral suppression for Haitians (neither were the indices using the overall population). Most striking was the lack of consistency on where socioeconomic status variables loaded highest among women, Hispanics, Haitians, and Non-Hispanic Whites. For these populations, socioeconomic status variables grouped together with two substance use variables (adherence to ARTs when using drugs and substance use treatment needs), creating factors that were hard to interpret. Overall, for Haitians, the variables considered as vulnerable/enabling indicators of health care behavior may differ or may need to be measured differently. In addition, the quality of communication between Haitian patients and case managers may have been suboptimal when obtaining data on some of these factors.

This study has two primary limitations. First the variables considered for the development of vulnerable and enabling indices were selected based on their availability in our dataset. Variables such as incarceration history, stigma, self-efficacy, and acceptance of diagnosis [5,6] were not in the datasets that were available for analysis. Second, as mentioned earlier, data were collected as part of service delivery protocols for the RWP. While this has advantages of being readily available and practical in the clinical setting, validated measurement instruments were not used, and data was collected by numerous case managers across multiple agencies in the county.

## 5. Conclusions

In summary, we developed vulnerable and enabling indices tailored for racially and ethnically diverse population of PLWH. Vulnerable and enabling factors fell into three constructs: mental health, drug use, and socioeconomic status. Using these or similar indices by clinicians can provide a more comprehensive assessment of the diverse dimensions affecting quality of care for PLWH. The internal reliability of the vulnerable/enabling variables used in the general indices was acceptable, and indices showed construct validity and predictive utility. However, the effect sizes were small. For the secondary objective, our results suggest that gender- (men/women) and/or race/ethnicity-specific (Non-Hispanic Black, Hispanic, Haitian, Non-Hispanic White) vulnerable/enabling indices are not needed. The general indices had similar validity and utility across gender and racial/ethnic diverse populations, except with Haitian PLWH.

## Figures and Tables

**Table 1:** Variables included in vulnerable/enabling indices and factor loadings from principal component analysis for overall indices and gender- and racial/ethnic-specific indices among Miami–Dade County Ryan White Program clients.

	Factor Loadings for Overall and Gender- and Racial/Ethnic-Specific Indices						
Indices	Overall	Men	Women	Non-Hispanic Black	Hispanic	Haitian	Non-Hispanic White
**Mental health index**							
Mental health symptoms	0.57669	0.57659	0.56891	0.51185	0.57715	0.49753	0.56770
Mental health services need	0.57670	0.57660	0.53185	0.53246	0.57715	0.46988	0.56775
Domestic violence	0.57669	0.57659			0.57715	0.50621	0.56761
**Drug and alcohol use index**							
Drug use	0.54951	0.55759	0.48632	0.48952	0.56547	0.56224	0.50277
Problematic drug use	0.47197	0.46680	0.43508	0.41685	0.47791	0.51128	0.50374
Non-adherence to ARV when using drugs	0.47606	0.49997		0.40569	0.51396	0.49325	
Substance use treatment needs							0.42140
**Socioeconomic status index or socioeconomic status and drug use index**							
Homelessness	0.47905	0.49325	0.43475	0.44550	0.52165	0.51271	0.43744
Not working	0.47167	0.46351	0.49603	0.42765	0.42472		
Non-adherence to ART when using drugs			0.56863				0.58112
Substance use treatment needs					0.42193	0.52965	
**Variables deleted**							
Domestic violence			^b^	^b^			
Substance use treatment needs	^b^	^b^	^b^	^b^			
Not working						^a^	^b^
No social support	^a^	^a^	^a^	^a^	^a^	^a^	^a^
Transportation need	^a^	^a^	^a^	^a^	^a^	^b^	^a^
Food insecurity	^b^	^b^	^a^	^b^	^b^	^a^	^b^
Perinatal HIV exposure	^a^	^a^	^a^	^a^	^a^	^a^	^a^
IDU exposure	^a^	^a^	^a^	^a^	^b^	^a^	^a^
Number of minors in the household	^a^	^a^	^a^	^a^	^a^	^a^	^a^
Non-disclosure	^a^	^a^	^a^	^a^	^a^	^a^	^a^
**Reliability analysis**							
Cronbach alpha							
Original ^c^	0.5699	0.5641	0.5869	0.5563	0.5409	0.4745	0.5881
Final ^d^	0.6752	0.6627	0.7071	0.6662	0.6655	0.5957	0.7150
**Percent variance accounted for by factors (%)**	79.2	79.1	71.3	68.6	72.4	59.1	85.3

^a^ Variables deleted because “Cronbach’s alpha if item deleted” was high; ^b^ Variables deleted because their factor loading was <0.4; ^c^ Includes all variables considered for analysis; ^d^ Excludes variables that increased the Cronbach’s alpha if item was deleted and variables with a factor loading of <0.4 in principal component analysis.

**Table 2:** Range of scores, and convergent validity and predictive utility for non-viral suppression of overall vulnerable/enabling indices for the Miami–Dade County Ryan White Program client population.

	Convergent Validity and Predictive Utility for Non-Viral Suppression						
Indices	Overall (N = 6939)	Men (N = 5330)	Women (N = 1609)	Non-Hispanic Black (N = 1731)	Hispanic (N = 3989)	Haitian (N = 747)	Non-Hispanic White (N = 472)
**Mental health index**							
Mean score (SD)	0 (1) ^a^	−0.05 (0.94)	0.15 (1.17)	0.23 (1.16)	−0.06 (0.93)	−0.33 (0.59)	0.19 (1.17)
Point-biserial correlation coefficient	0.11 ***	0.13 ***	0.08 **	0.10 ***	0.11 ***	0.02	0.12 *
Odds ratio (95% CI)	1.31 (1.24–1.39)	1.37 (1.28–1.47)	1.18 (1.06–1.31)	1.20 (1.10–1.31)	1.37 (1.26–1.50)	1.11 (0.82–1.50)	1.31 (1.07–1.62)
**Drug and alcohol use index**							
Mean score (SD)	0 (1) ^a^	0.01 (1.02)	−0.04 (0.94)	0.23 (1.33)	−0.08 (0.84)	−0.20 (0.56)	0.13 (1.18)
Point-biserial correlation coefficient	0.14 ***	0.14 ***	0.13 ***	0.14 ***	0.12 ***	0.08 *	0.13 *
Odds ratio (95% CI)	1.33 (1.26–1.40)	1.34 (1.26–1.42)	1.31 (1.18–1.47)	1.23 (1.14–1.33)	1.35 (1.24–1.47)	1.33 (1.02–1.74)	1.28 (1.07–1.54)
**Socioeconomic status index**							
Mean score (SD)	0 (1) ^a^	−0.04 (1.01)	0.13 (0.96)	0.41 (1.19)	−0.18 (0.85)	−0.02 (0.92)	0.05 (1.05)
Point-biserial correlation coefficient	0.17 ***	0.19 ***	0.12 ***	0.15 ***	0.17 ***	0.06	0.12 *
Odds ratio (95% CI)	1.50 (1.42–1.58)	1.54 (1.45–1.64)	1.34 (1.18–1.51)	1.31 (1.20–1.43)	1.63 (1.48–1.79)	1.18 (0.98–1.43)	1.33 (1.06–1.67)

CI, confidence interval; SD, standard deviation; ^a^ Scores standardized based on overall population. Overall population mean scores are zero and standard deviation one. Higher scores correspond to worsening mental health, drug use, or socioeconomic status depending on the index. * *p*-value < 0.05; ** *p*-value < 0.001; *** *p*-value < 0.0001.

**Table 3:** Range of scores, convergent validity, and predictive utility for non-viral suppression of gender- and racial/ethnic-specific vulnerable/enabling indices for the Miami–Dade County Ryan White Program client population.

	Convergent Validity and Predictive Utility for Non-VIRAL Suppression					
Indices	Men (N = 5330)	Women (N = 1609)	Non-Hispanic Black (N = 1731)	Hispanic (N = 3989)	Haitian (N = 747)	Non-Hispanic White (N = 472)
**Mental health index ^a^**						
Point-biserial correlation coefficient	0.13 ***	0.08 **	0.10 ***	0.11 ***	0.02	0.12 *
Odds ratio (95% CI)	1.35 (1.26–1.44)	1.23 (1.09–1.39)	1.25 (1.12–1.39)	1.34 (1.24–1.46)	1.06 (0.89–1.27)	1.37 (1.08–1.75)
**Drug and alcohol use ^a^ index**						
Point-biserial correlation coefficient	0.14 ***	0.16 ***	0.13 ***	0.12 ***	0.08 *	0.19 ***
Odds ratio (95% CI)	1.34 (1.27–1.43)	1.35 (1.23–1.49)	1.32 (1.20–1.46)	1.29 (1.20–1.38)	1.17 (1.01–1.36)	1.45 (1.19–1.78)
**Socioeconomic status index or socioeconomic status and drug use index ^a^**						
Point-biserial correlation coefficient	0.19 ***	0.11 ***	0.14 ***	0.15 ***	0.10 *	0.10 *
Odds ratio (95% CI)	1.55 (1.45–1.66)	1.26 (1.13–1.41)	1.37 (1.23–1.53)	1.35 (1.26–1.46)	1.23 (0.99–1.52)	1.25 (1.00–1.58)

CI, confidence interval; SD, standard deviation; ^a^ Scores standardized based on gender/racial/ethnic specific population. Gender/racial/ethnic specific population mean scores are zero and standard deviation one. * *p*-value < 0.05; ** *p*-value < 0.001; *** *p*-value < 0.0001.

## Data Availability

Not applicable.
